# Nurses’ Experience Implementing an Automated Video Monitoring System to Decrease the Risk of Patient Falls during a Global Pandemic

**DOI:** 10.3390/healthcare11182556

**Published:** 2023-09-15

**Authors:** Joseph A. Allen, Roni Reiter-Palmon, Katherine J. Jones, Lucas Sabalka, Kelsey Ciagala, Andrea Meens

**Affiliations:** 1 Department of Family and Preventive Medicine, School of Medicine, University of Utah, Salt Lake City, UT 84111, USA; 2 Department of Psychology, College of Arts and Sciences, University of Nebraska at Omaha, Omaha, NE 68182, USA; rreiter-palmon@unomaha.edu (R.R.-P.); kciagala@unomaha.edu (K.C.); 3 Nebraska Coalition for Patient Safety, Omaha, NE 68198, USA; katherine.jones@volunteer.unmc.edu; 4 Ocuvera, Lincoln, NE 68512, USA; lsabalka@ocuvera.com (L.S.); ameens@ocuvera.com (A.M.)

**Keywords:** technology, patient safety, social influence, COVID-19, video

## Abstract

Healthcare is a complex sociotechnical system where information systems (IS) and information technology (IT) intersect to solve problems experienced by patients and providers alike. One example of IS/IT in hospitals is the Ocuvera automated video monitoring system (AVMS), which has been implemented in more than 30 hospitals. The purpose of this study was to evaluate nurses’ attitudes toward AVMS implementation over time as they received the training program developed for this intervention. Consistent with the job demands–resources (JDR) model, we found that perceptions of AVMS usefulness increased over time and were positively associated with perceptions of social influence and behavioral control. These results were consistent with our finding that there was a significant decrease in the risk of unassisted falls from the bed from baseline to intervention. Leaders in hospital systems and healthcare organizations may want to consider implementing an AVMS as researchers continue to test, verify, and demonstrate the effectiveness of these interventions for improving patient well-being.

## 1. Introduction

Healthcare is a sociotechnical system; human beings work in social structures within complex technical environments to achieve large, complex goals [1]. The use of information systems (IS) and information technology (IT) in the medical and healthcare field has increased during the past few decades. One example of IS/IT in hospitals is the Ocuvera automated video monitoring system (AVMS), which has been implemented in more than 30 hospitals. The AVMS monitors a patient’s movement, uses machine learning to identify when that movement is consistent with the patient preparing to exit their bed or chair, and sends a video alert to nurses via mobile devices and displays at each nurses’ station. Previous research determined that the median response time for the AVMS, which is the time between the video alert beginning and a nurse responding to the patient, was 28.5 s. This lead time was associated with an 89% decrease in the rate of unattended bed exits per day and a 78% decrease in the risk of injurious falls [2]. IS/IT use, including the AVMS, has been proposed to improve patient care, decrease overall costs, and improve workflow. Implementing these systems is often costly and time-consuming, and the endeavor may end in failure if too little attention is paid to the social structure—the knowledge, attitudes, skills, and resources—of the people who are intended to use the IS/IT. 

Implementing the AVMS in a hospital system is a large-scale organizational innovation. Successful implementation of innovations requires that the innovation be consistent with the values of the organization, be supported by management and frontline champions, have financial support, and have an explicit implementation plan. This plan should include needed modifications to the organization’s equipment/environment; clarification of roles and tasks (i.e., job demands); and changes to policies, procedures, and job descriptions to routinize the innovation [3]. Job demands include the time and effort needed for frontline staff to learn to use the innovation, for managers to champion it and provide feedback about its emerging use, and for administrators to change organizational structures to routinize it [3]. 

The job demands–resources (JD-R) model is an occupational stress model [4]. This model suggests that when job demands increase, the resources to meet those demands must increase correspondingly or employees will exhaust their physical, emotional, and social resources. This exhaustion may lead to employee burnout and failed implementation of an innovation [5]. However, when an intervention transitions from a new demand to a meaningful resource, the intervention may be perceived as successful rather than stressful.

The purpose of this study was to evaluate nurses’ attitudes toward AVMS implementation over time in two units of a Midwestern acute care hospital after a customized training program. The development of the training program and initial work with the hospital started in January 2020. The training program and AVMS implementation occurred from October 2020 through September 2021. The COVID-19 pandemic began in March 2020 and ended as a public health emergency in the U.S. in May 2023 [6]. Because we sought to implement the AVMS during the COVID-19 pandemic when job demands within hospitals stressed organizational resources, we used the JD-R model as a framework to evaluate nurses’ perceptions of the usefulness of the AVMS as well as the social support and resources needed to adopt the AVMS into practice. By demonstrating the potential usefulness of the AVMS in reducing patient falls while also inspiring those who implement the technology, we contribute to the ongoing efforts to decrease the risk of inpatient falls, also known as a serious problem in healthcare [7].

What we did not expect as we planned the current study was the onset of a global pandemic, specifically COVID-19. The pandemic abruptly forced the healthcare system into action to mitigate the effects and outcomes of an unpredictable disaster that unfolded over the course of the entire life of this project. Thus, we provide context where appropriate concerning the learnings from both the research itself and the challenges to the implementation of this study. 

### 1.1. Patient Falls and Video Monitoring

Prior to investigating the main purpose of the current study, it is important to establish once again that the AVMS decreases the risk of falls. Specifically, previous work with the AVMS showed that fall rates are reduced when the AVMS is implemented [2]. Before we can truly evaluate nurses’ experience using the AVMS, we need to first establish that the current study includes a similar situation and set of results. That is, the AVMS should reduce fall rates in our study, and the attitudes observed should be representative of those likely experienced by other groups that implemented the AVMS. The Ocuvera system is designed to alert nurses of patient behavior only when the patient is unassisted and only when the patient’s movement is consistent with attempting to exit their bed or chair unassisted. At the time of this study, the Ocuvera system only monitored patient movement in the bed (support for falls originating from the chair was introduced in December 2021). Hence, we focus on targeted falls as unassisted falls originating from the bed. Therefore, our first hypothesis, which is a partial replication of previous work [2], is as follows:

**Hypothesis** **1.** *Implementing the AVMS will be associated with a decreased risk of unassisted falls where the patient originates from the bed (“targeted falls”).*

### 1.2. Training and Nurse Use of New Technology

Successful implementation of new technology requires that users understand the technology, be able to use it successfully, and believe that it will be useful [8]. To ensure that nurses were able to use the newly implemented technology and understood its purpose and usefulness, we developed a training program in which nurses were able to learn about the technology and practice its functions. Importantly, the act of using the technology over time can also influence perceptions regarding the technology. With successful use, perceptions of usefulness are likely to increase over time.

To address the purpose of evaluating nurses’ attitudes towards the AVMS over time, we propose three hypotheses:

**Hypothesis** **2.** *Perceptions of the usefulness of the AVMS will improve over time during intervention*.

**Hypothesis** **3.** *Perceptions of social influence support for AVMS will improve over time.*

**Hypothesis** **4.** *Perceptions of social influence support and behavioral control will be positively associated with perceptions of the usefulness of the AVMS during intervention*.

## 2. Methods

### 2.1. Nurse Training Programs

There were two phases in this study. The baseline phase occurred 1 January–19 November 2020. This phase included implementing the AVMS in two nursing units, developing and implementing two adaptive training programs to teach nurses how to integrate the AVMS into their existing workflows, and collecting fall-related data. The AVMS was activated on 27 July 2020. At this time, nurses began providing informed consent to eligible patients who chose to participate. Patient movement was recorded, but nurses did not receive alerts on mobile devices or at the central monitoring station. The intervention phase occurred 20 November 2020–30 September 2021. During this phase, nurses received alerts on mobile devices and at the central monitoring station. They continued to provide informed consent to patients who chose to participate, and we continued to collect fall-related data.

The first introductory training sessions were conducted on 7–29 October 2020. The main goals of this training were to teach nurses to correctly apply the eligibility criteria (i.e., patients assessed to be at high risk of falls), educate nurses on proper use of the system, and facilitate technology adoption (a key barrier identified in prior work [2]). We conducted the training program on the hospital’s training day for all nurses who might care for patients in the two study units. Nurses took the training in groups ranging from three to six individuals. The training consisted of a PowerPoint presentation in which we introduced the concept of the AVMS and explored how the technology worked and why it is an effective tool for reducing unattended bed exits. This presentation was followed by a five-minute training video developed by the researchers. The video detailed how to work the various aspects of the AVMS using audio and visual information. After the video, an Ocuvera representative performed a live demonstration of the technology. Only the Ocuvera representative handled the technology, and the nurses were not allowed to practice due to concerns about COVID-19. After the training, the nurses were asked to complete a survey that included various reactions to and perceptions of the Ocuvera system along with knowledge questions to evaluate the effectiveness of the nurse training. 

Charge nurses were asked to take a second, more in-depth AVMS training two months after the introductory training. This training, labeled the superuser training, aimed to give charge nurses a more detailed explanation of how the technology worked and common troubleshooting techniques, allowing them to use the technology more effectively and efficiently. This training was virtual and consisted of a PowerPoint presentation and an opportunity for charge nurses to ask questions or raise concerns. The training was conducted by the researchers along with a representative from Ocuvera. 

### 2.2. Participants and Procedure

The survey was administered in three waves over the course of the project. Wave 1 took place in October 2020 (*n* = 51) and was administered via iPads immediately after nurses received the introductory training at the hospital’s annual review of skills. Waves 2 and 3 took place in June (*n* = 37) and October 2021 (*n* = 30) and were administered via iPads during mandatory monthly nurse meetings. Due to COVID-19 restrictions, the superuser training was administered three months into the use of the AVMS and included fewer charge nurses than intended. Further, researchers and Ocuvera representatives were not allowed onsite in the first month to support implementation due to these restrictions. We estimate that about 80% of patients at high risk of falling (i.e., eligible for inclusion) were monitored using the AVMS. However, we also estimate that only about 40% of patient days for patients at high risk were monitored. 

There were a number of barriers to the intervention due to the COVID-19 pandemic that we had to overcome in order to have any success in the adoption of the AVMS and the learning needed from the training. First, we had to transition the superuser training from an in-person, hands-on training to a virtual training. Second, we had to lean more heavily upon our onsite champions to represent the interests of the intervention. This translated into many more emails, phone calls, and virtual meetings with the onsite leadership than originally expected. Third, whenever there was a lull in COVID-19 infections, we made efforts to increase our visibility at the site. This included conducting interviews and engaging with participants to gauge their experiences. We acknowledge that these efforts likely affected nurses’ experience with social influence and behavioral control in this study, though we did not measure these specific additional intervention behaviors. 

### 2.3. Measures

Perceived usefulness of the Ocuvera system was measured via five items on a scale of 1—strongly disagree to 5—strongly agree (*α* = 0.930) [9,10]. Items include these samples: “The Ocuvera system will improve patient safety on study units” and “The Ocuvera system will make my job easier on study units”. 

Perceived ease of using the Ocuvera system was measured via four items on a scale of 1—strongly disagree to 5—strongly agree (*α* = 0.880) [10,11,12]. Items include this sample: “The Ocuvera system will be easy to use”. 

Perceived behavioral control over using the Ocuvera system was measured via four items on a scale of 1—strongly disagree to 5—strongly agree (*α* = 0.795) [9,13]. Items include this sample: “The hospital has the necessary resources to use the Ocuvera System”.

Perceived social influence related to using the Ocuvera system was measured via five items on a scale of 1—strongly disagree to 5—strongly agree (*α* = 0.836) [9,11,13]. Items include this sample: “People who are important to me think I should use the Ocuvera system”. 

Nurses’ attitudes towards falls were measured via four items on a scale of 1—strongly disagree to 5—strongly agree (*α* = 0.601) modified from Miake-Lye and colleagues [14]. Items include this sample: “We can prevent most patient falls”.

Further, questions about nurses’ perceptions of the impact of the COVID-19 pandemic on the implementation of the AVMS were added to waves 2 and 3. Items were rated on a scale from 1—not a lot to 5—a great extent and example questions include “Implementing the Ocuvera system was not a priority when we were at high census due to COVID-19” and “The Ocuvera system helped us to care for patients with COVID-19 because we could see the patients on the mobile device or monitoring station without entering the room”. The evaluation of nurses’ attitudes towards the AVMS system was approved by the Institutional Review Board of the university associated with this study, which has been blinded in this paper. 

The primary outcome used to determine the effectiveness of the AVMS as a quality improvement project for the hospital was the rate of targeted falls per 1000 patient days. A secondary outcome was the rate of all unassisted falls per 1000 patient days. Consistent with the Agency for Healthcare Research and Quality [15], the hospital defined a fall as “a sudden, unintended, descent of a patient’s body to the ground or other object (e.g., onto a bed, chair, or bedside mat) that can be assisted or unassisted”. We determined the numerators (numbers of falls) for these rates from aggregate deidentified fall-related data. We determined the denominator of patient days from deidentified administrative data.

## 3. Results

Prior to testing the hypotheses, preliminary analyses including means, standard deviations, and correlations were computed and are provided in Table 1.

Hypothesis 1 stated that implementing the AVMS will be associated with a decreased risk of targeted falls within the study units. Comparing baseline to intervention phases using the general test statistic, targeted falls per 1000 patient days decreased from 1.71 to 1.0 (*p =* 0.007). All unassisted falls per 1000 patient days decreased from 3.04 to 2.28 (*p =* 0.072). These findings provide preliminary support for H1.

T-tests were used to test hypotheses 2 and 3. Hypothesis 2 stated that perceptions of the usefulness of the AVMS will improve over time. To test this hypothesis, we compared the perceived usefulness of the AVMS in waves 2 and 3, since wave 1 served as a baseline and no interaction with the system had yet occurred. Results indicated that the AVMS was perceived as more useful in wave 3 than in wave 2, *t*(10) = −4.74, *p* < 0.05, providing support for H2.

Hypothesis 3 stated that perceptions of social influence support for the AVMS will improve over time. To test this hypothesis, we compared the social influence support for the system from wave 1, before the system was running, to wave 3, after the system had been in use for a year. Nurses perceived that social influence such as support from upper management and their coworkers to use the AVMS was greater in wave 3 than in wave 1, *t*(9) = −4.34, *p* < 0.05, providing support for H3.

Hypothesis 4 stated that perceptions of social influence support and behavioral control will be positively associated with perceptions of the usefulness of the AVMS during intervention. To test this hypothesis, we used regression analyses using data from the last two timepoints, regressing both social influence and behavioral control onto perceptions of AVMS usefulness while controlling for attitudes towards falls. At time two, only social influence (*β* = 0.39, *p* < 0.05) related to usefulness, and at time three, only behavioral control (*β* = 0.41, *p* < 0.05) related to usefulness (see Table 2 and Table 3). These findings provide partial support for Hypothesis 4. However, it should be noted that at times two and three, behavioral control and social influence were both approaching significance, and the limiting factor was the reduction in power to detect the effect. We discuss this further in the Section 4.

## 4. Discussion

We sought to evaluate nurses’ attitudes towards AVMS implementation over time in two Midwestern acute care hospital units after a customized training program. Consistent with the JDR model, we found that perceptions of AVMS usefulness increased over time and were positively associated with perceptions of social influence and behavioral control. These results are consistent with our finding that there was a significant decrease in the risk of unassisted falls from the bed from baseline to intervention.

Our findings are consistent with theoretical frameworks regarding the social and organizational resources needed to implement an IS/IT intervention as an organizational innovation [16]. In fact, in a review of nurses’ attitudes towards use of new technologies, Kaye found that nurses’ attitudes in relation to the ease of use and usefulness of a given implementation were essential to any IS/IT intervention. Thus, finding that the AVMS is both clinically effective at reducing falls and viewed as useful, particularly when social influence support and behavioral control resources are available, makes the current study even more meaningful. 

Consistent with previous studies of the effectiveness of the AVMS [2], we found that its implementation was associated with a significant decrease in the risk of unassisted falls originating from the bed. This finding is surprising since multiple studies have reported an increase in the risk of inpatient falls during the COVID-19 pandemic. This heightened risk was due to patient factors such as an increased prevalence of comorbidities [17] and system factors such as nursing staff shortages [18] and delayed response time to meet patient needs as nursing staff donned personal protective equipment before entering a room [17].

We were so concerned about this possibility that we added a question to the postsurvey concerning the nature of the intervention in terms of priority. We found that, during the height of the COVID-19 pandemic at the study hospital, many nurses (37.8% in wave 2 and 36.7% in wave 3) indicated that implementing the AVMS was not a priority. Rightfully so, the priority was caring for the influx of patients with COVID-19, which increased the demands on the entire hospital and healthcare workers overall. Because attention was pulled from the implementation of the AVMS, we expect that the findings reflect the most conservative test of the new system to date. With nurses’ attention diverted to caring for patients during a global pandemic, the training associated with the AVMS and the new procedures required for the AVMS were often ignored. In fact, the research team spent much of its efforts during the implementation encouraging the key champions to communicate the potential usefulness of the AVMS during a pandemic, which included the ability to remotely monitor the movement of patients who tended to be sicker. The research team expected that the findings would be relatively weak given the interruption of the protocol by required changes in practice to manage the pandemic. The fact that the findings nonetheless demonstrated a decrease in the risk of targeted falls is a testament to the resilience of the nursing staff and research team and the effectiveness of the AVMS to improve patient safety and nurses’ experiences at work.

### 4.1. Implications for Research

The study presented here has several implications for research. First, the JD-R framework is useful both in understanding the hypotheses tested and in understanding how the intervention worked, even during a pandemic. For instance, the pandemic created an abrupt and extended job demand that pulled attention, resources, and time away from the intervention. However, the success of the intervention at reducing targeted falls, as well as being perceived as useful, likely helped provide resources back into the system, making the intervention effective despite the challenging context. Thus, JD-R was useful from a theoretical perspective in explaining why and how the hypotheses worked during the intervention occurring in a crisis. 

Second, the study demonstrated that when an intervention is effective in achieving key job demands during regular operations, it can remain effective when those operations are stressed. From a researcher perspective, these findings are a reminder that studies should not be abandoned under challenging circumstances if meaningful adjustments can be made to successfully complete them. Our adjustments included wearing PPE, delivering training remotely, communicating with champions virtually instead of in person, and pressing forward despite calls to pause the intervention. Because previous studies supported the use of video monitoring in general, we felt it would be unethical to stop the intervention given its likelihood of making a positive impact on patient well-being.

Third, training that includes special augmented components for superusers of an intervention appears to be a meaningful step that researchers should consider using more frequently. Work on training shows the usefulness of instruction to transfer knowledge and skills to others and even to change behavior when implemented effectively [19]. However, the use of augmented training for people who show particular interest in the intervention has not been used extensively in intervention science. The findings suggest that perhaps there are some benefits to having enhanced training for such users, who often include the intervention champions.

### 4.2. Practical Implications

Despite the increased job demands of providing care for highly infectious patients, nurses were able to integrate the AVMS into their workflow over time as social support and resources improved. Further, the training we provided may serve as a meaningful pattern for use in implementing this intervention and perhaps others. Additionally, it appears as though support from leadership in the form of both social influence and the resources needed for behavioral control may enhance the usefulness of the intervention.

As another practical implication, the unfortunate truth about patient falls is that they can cause injury and open up the healthcare organization to potential legal issues [20]. Given these practical challenges that falls present, implementing AVMS appears to be a reasonable response. Furthermore, healthcare managers and leaders should consider other tools, such as an incident reporting system, to help both mitigate and learn from patient falls [20]. The key to reducing patient falls appears to be learning from them, in some cases. 

### 4.3. Limitations and Future Directions

Despite the meaningful nature of the findings, this study was not without limitations. First, it was implemented during the COVID-19 pandemic. Adjustments were made throughout to mitigate the problems introduced by the pandemic, which we did not anticipate when planning the study. Despite the challenges, the findings indicate that the intervention worked. However, we are not confident that the effects shown here are accurate to what one might see if this intervention occurred outside of a crisis situation. Future research is needed to determine the actual magnitude of the effect on both patient falls and nurses’ experiences.

Second, the findings, particularly in relation to Hypothesis 4, were greatly impacted by the small sample size, which reduced overall statistical power. All the hypothesized effects were in the directions predicted, but the small sample size likely kept us from finding significant relationships. The attrition was likely due to competing demands originating from the COVID-19 pandemic. Several nurses who initially engaged in this study simply were not able to continue in the study as the pandemic ebbed and flowed at the study site. Additionally, the small sample size limits the ability to generalize our findings to the population of nurses in a variety of other settings. Thus, future research should expand the collection approach to verify and expand upon the current findings.

Third, this study had the potential for researcher and participant bias entering into the process. To mitigate this concern, the researchers only interacted with the nurses during the required trainings. Circumstances did not allow check-ins (COVID-19), which actually served to mitigate the influence of the researchers on the methods and results. In terms of participants, we did not include the site leader (i.e., intervention champion) data in the analyses, thus removing one potentially biased participant. However, others could have been biased by association, so this concern remains, and future research needs to introduce mechanisms to mitigate the concern.

Fourth, the study site is unique to the location, both in terms of implementation parameters as well as how the COVID-19 pandemic unfolded. As such, the findings are not as generalizable as we would prefer. Thankfully, this provides an opportunity to test these hypotheses once more to both increase generalizability and hopefully identify the actual size of the effect of the intervention (see the first limitation). 

Fifth, the study design did not have a comparative group (i.e., control group), thereby limiting the strength of the conclusions and findings. We recommend readers interpret the findings with caution due to this and other limitations. However, we hope that the findings and potential implications inspire future research where a randomized controlled trial could occur, including a control condition and a comparative technology group. Taking that approach would expose the benefits of AVMS more fully and help to identify key features of the technology that drive the findings. 

## 5. Conclusions

The purpose of this study was to evaluate nurses’ perception and evaluation of an AVMS over time. The results indicated that usefulness perceptions increased over time and were related to perceptions of social influence and behavioral control, as predicted by the JD-R model. In addition, we found that over time, there was a significant decrease in the risk of unassisted falls originating from the bed. The results of this study are particularly meaningful as the sample size was small, leading to low power and lower likelihood of finding significant results. In addition, the fact that the study took place during the height of the COVID-19 pandemic suggests that the results may be conservative and that we may find stronger effects when hospitals are under normal operations.

## Figures and Tables

**Table 1:** Means, standard deviations, and correlations for study variables.

Measure	M	*SD*	1	2	3	4	5	6	7	8	9	10	11	12	13	14
1. Usefulness time 1	3.56	0.76	-													
2. Social influence time 1	3.76	0.71	0.54 *	-												
3. Behavioral control time 1	3.70	0.74	0.58 *	0.46 *	-											
4. Fall attitudes time 1	3.99	0.59	0.39 *	0.33 *	0.34 *	-										
5. Usefulness time 2	3.36	0.95	0.48 *	0.54 *	0.42 *	0.07	-									
6. Social influence time 2	3.95	0.83	0.34 *	0.61 *	0.44 *	0.36 *	0.58 *	-								
7. Behavioral control time 2	3.61	0.86	0.18	0.44 *	0.31	0.24	0.59 *	0.63 *	-							
8. Fall attitudes time 2	3.79	0.61	0.10	0.06	0.07	0.10	0.20	0.16	0.20	-						
9. Usefulness time 3	3.54	0.71	0.31	0.38	0.37	0.10	0.49 *	0.67 *	0.45 *	−0.28	-					
10. Social influence time 3	4.04	0.59	0.11	0.54 *	0.10	−0.04	0.41	0.52 *	0.55 *	−0.15	0.63 *	-				
11. Behavioral control time 3	3.82	0.67	0.15	0.25	−0.09	−0.13	0.23	0.55 *	0.51 *	−0.33	0.48 *	0.67 *	-			
12. Fall attitudes time 3	3.76	0.54	0.06	0.16	0.20	0.45 *	−0.09	−0.29	−0.06	0.60 *	0.09	0.28	0.21	-		
13. Gender	1.89	0.31	0.09	0.02	0.01	0.00	0.09	0.08	−0.01	−0.14	0.51 *	0.16	0.05	0.14	-	
14. Age	33.81	9.31	−0.02	−0.05	0.02	−0.12	−0.11	−0.06	−0.10	−0.29	−0.08	−0.09	0.17	−0.14	0.03	-

Note: * p < 0.05.

**Table 2:** Regression of AVMS usefulness perceptions in wave 2.

Model	*B*	*SE*	*t*	*F*	*R* ^2^
Intercept	1.20	0.77	1.55	7.43	0.30
Social influence	0.39 *	0.16	2.42		
Behavioral control	0.24	0.15	1.52		
Fall attitudes	0.05	0.17	0.32		

Note: *n* = 34. * *p* < 0.05.

**Table 3:** Regression of AVMS usefulness perceptions in wave 3.

Model	*B*	*SE*	*t*	*F*	*R* ^2^
Intercept	1.17	0.87	1.34	5.44	0.27
Social influence	0.28	0.23	1.20		
Behavioral control	0.41 *	0.20	2.00		
Fall attitudes	−0.03	0.19	0.00		

Note: *n* = 26. * *p* < 0.05.

## Data Availability

Anonymized data are available upon request of the authors and in accordance to IRB protocol provisions.
